# Vacuum-Assisted Percutaneous Management of Cardiac Implantable Electronic Device Lead Endocarditis

**DOI:** 10.3390/jcm15114276

**Published:** 2026-06-01

**Authors:** Robertas Pranevičius, Rasa Ordienė, Sandra Kmitaitė, Agnė Rimkutė, Rugilė Kairaitytė, Ramūnas Unikas

**Affiliations:** 1 Heart Centre, Lithuanian University of Health Sciences, 44307 Kaunas, Lithuania; robertas.pranevicius@lsmu.lt (R.P.); rasa.ordiene@lsmu.lt (R.O.); rugile.kairaityte@kaunoklinikos.lt (R.K.); ramunas.unikas@lsmu.lt (R.U.); 2 Faculty of Medicine, Medical Academy, Lithuanian University of Health Sciences, 44307 Kaunas, Lithuania; sandra.kmitaite@stud.lsmu.lt

**Keywords:** CIED infection, infective endocarditis, Penumbra system, percutaneous aspiration, vacuum-assisted thrombectomy, ICD lead vegetation, minimally invasive treatment

## Abstract

**Background and Clinical Significance:** Infective endocarditis is a disease of the endocardial surface of the heart, most often affecting heart valves (native or prosthetic) or intracardiac device. Although relatively rare, it carries high embolic risk of complications and mortality. Complete device extraction is recommended; however, conventional surgery may be prohibitive in frail patients with multiple comorbidities. **Case presentation:** We present a case of a 74-year-old male with implantable cardioverter-defibrillator (ICD)-related infective endocarditis and large lead-associated vegetation measuring approximately 3 cm in size. Due to a high operative risk assessed by Euro SCORE II, a minimally invasive percutaneous approach using the Penumbra vacuum-assisted aspiration system was selected. The procedure enabled successful debulking of the vegetation, followed by complete device removal and targeted antibiotic therapy. The patient’s clinical condition improved, with normalization of inflammatory markers and no recurrence of infection, and a new ICD was safely reimplanted after recovery. **Conclusions:** This case highlights the potential role of percutaneous vacuum-assisted aspiration as an effective and less invasive therapeutic option in high-risk patients with CIED-related infective endocarditis, particularly when conventional surgical management is contraindicated or requires bridging therapy until the patient’s status is stabilized.

## 1. Introduction

Infective endocarditis is an infection of the inner lining of the heart muscle (endocardium). Bacteria, fungi or other germs that enter through the bloodstream can cause vegetative masses on heart valves, or these can be cardiac device-related [1]. According to the 2023 European Society of Cardiology (ESC) Guidelines, the global incidence of IE is estimated at approximately 13.8 cases per 100,000 persons annually, accounting for nearly 66,300 deaths worldwide in 2019. Despite significant advances in diagnostic imaging, microbiology, and surgical management, in-hospital mortality remains high, ranging from 15% to 30% [1,2,3]. Prosthetic valve endocarditis (PVE) currently accounts for approximately 20–30% of all IE cases and is associated with significantly worse outcomes compared with native valve endocarditis. The reported incidence of PVE among patients with prosthetic valves ranges from 0.3% to 1.2% per patient-year [4,5,6]. Cardiac implantable electronic device-related infective endocarditis (CIED-IE) represents one of the fastest-growing subgroups of IE due to the expanding use of pacemakers, implantable cardioverter-defibrillators (ICDs), and cardiac resynchronization therapy (CRT) devices. Device-related infection is considered one of the most serious complications of CIED therapy and is associated with substantial morbidity and mortality. The majority of CIED infections are caused by *Staphylococcus aureus* or coagulase-negative staphylococci. Heart failure remains the most common complication of left-sided infective endocarditis and the leading indication for urgent cardiac surgery. Studies cited in the ESC Guidelines report a heart failure prevalence ranging from 19% to 73% among patients with left-sided IE. Embolic events are also frequent, particularly in patients with large vegetations [7,8,9,10].

According to the European Society of Cardiology (ESC) 2023 Endocarditis treatment guidelines a complete cardiac implanted electronic device (CIED) removal is recommended for all patients with confirmed infection of the lead(s), as conservative treatment is associated with increased mortality due to the embolic risk [11,12]. Antimicrobial treatment forms the cornerstone of therapy. When the causative microorganism is unknown, patients often receive empirical antibiotics right away. Commonly, vancomycin combined with gentamicin is used, though broader-spectrum options like meropenem and linezolid may be considered in critical situations [13]. Once culture results are available, therapy is adjusted. Viridians group streptococci usually respond to penicillin G or ceftriaxone, with the addition of gentamicin in the first week. When the causative agent is *Staphylococcus aureus*, treatment is initially with oxacillin. If clinical improvement does not occur or if methicillin-resistant strains are detected, a switch to vancomycin is recommended. In the case of enterococcal infections, it is preferable to combination ampicillin with ceftriaxone or gentamicin, according to the specific sensitivity response profile. Yet antibiotics alone do not always suffice. However, certain patients are considered unsuitable for surgical operation, as the operative risk is excessively high due to their advanced age, multiple comorbidities and compromised overall health, leading to a high Euro Score II score [14]. In such cases, percutaneous minimally invasive techniques—such as vacuum-assisted lead vegetation removal—represent a valuable therapeutic alternative. These procedures offer reduced perioperative morbidity and mortality, shorter recovery times, and the possibility of infection control in patients who would otherwise be inoperable [15,16]. Consequently, vacuum-assisted debulking can serve as a life-saving intervention and represents a significant advancement in the management of CIED-related infective endocarditis in high-risk populations.

## 2. Case Presentation

We present a case of a 74-year-old male with a medical history significant for ischemic heart disease, dyslipidemia, pulmonary hypertension, diabetes mellitus, and stage three chronic kidney disease (CKD). In 2017, he experienced a myocardial infarction (MI) that necessitated coronary artery bypass grafting (CABG) to prevent future occurrences. The patient remained stable until 2019, when he suffered cardiac arrest due to ventricular fibrillation, which required percutaneous coronary intervention (PCI) involving angioplasty and stenting. Following this incident, the Heart Team recommended the implantation of an implantable cardioverter-defibrillator (ICD), which was subsequently performed. The patient was on optimal medical therapy for heart failure and ischemic heart disease, and routine follow-ups demonstrated no cardiac complications. In February 2023, progressive knee arthrosis led to a knee arthroplasty with the placement of a prosthetic implant. In November, the patient was admitted with an unidentified infection of the elbow, presenting with edema, pain, and erythema. Despite prior antibiotic treatment, the C-reactive protein (CRP) level had increased from 63 to 92 mg/L. An ultrasound-guided arthrocentesis was performed to facilitate culture growth and histological evaluation. *Staphylococcus epidermidis* was identified, prompting a switch to oxacillin, which effectively reduced the CRP level (from 92.1 to 74.3 mg/L) and alleviated symptoms. Two months later, the patient was readmitted with persistent inflammation of the elbow and knee, as well as fever. A consultation with the Rheumatology department was initiated, where blood tests and cultures confirmed a diagnosis of sepsis. The initial workup included routine tests, such as X-ray and electrocardiogram, and did not reveal any specific abnormalities. Blood tests demonstrated leukocytosis and elevated inflammatory markers, including a CRP level of 93 mg/L. Multiple blood cultures obtained during hospitalization yielded *Staphylococcus epidermidis*, with initial microbiological evaluation revealing Gram-positive cocci in clusters. The isolated microorganism demonstrated susceptibility to oxacillin and gentamicin, prompting initiation of targeted antibiotic therapy (Table 1).

**Figure 1:**
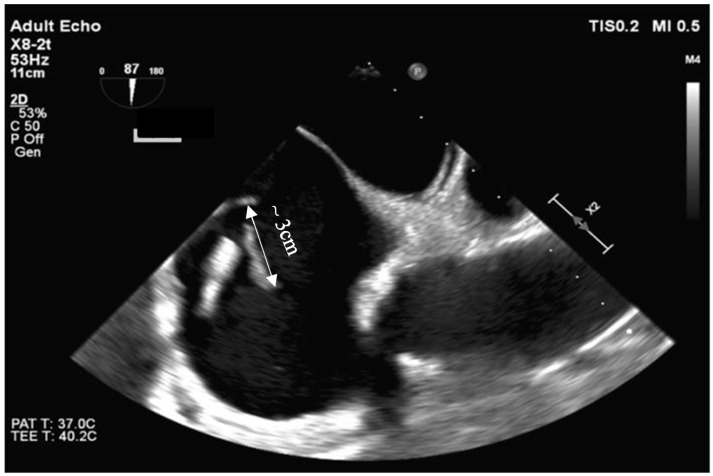
Transesophageal echocardiography demonstrating a ~3 cm vegetation on the ICD lead.

A computed tomography (CT) scan of the left elbow joint demonstrated lesions consistent with synovial chondromatosis and grade III arthrosis. The CT scan showed no significant fluid deposits. Transthoracic echocardiography (TTE) revealed a vegetation approximately 3 cm in size on the ICD lead, exhibiting mobility between the right ventricle and atrium, thereby posing a high embolic risk. For enhanced accuracy, a subsequent transesophageal echocardiography (TEE) was conducted and confirmed the presence of vegetation (~3 cm) on the implantable cardioverter-defibrillator (ICD) leads (Figure 1). The diagnosis of definite infective endocarditis was established according to the modified Duke criteria. Two major criteria were fulfilled: (1) positive blood cultures for *Staphylococcus epidermidis* obtained during the septic episode and (2) echocardiographic evidence of infective endocarditis demonstrated by TTE and confirmed by TEE showing a mobile vegetation measuring approximately 3 cm attached to the ICD lead. Minor criteria included fever exceeding 38 °C and the presence of a predisposing intracardiac device (ICD). Therefore, the patient fulfilled the criteria for definite infective endocarditis.

**Figure 2:**
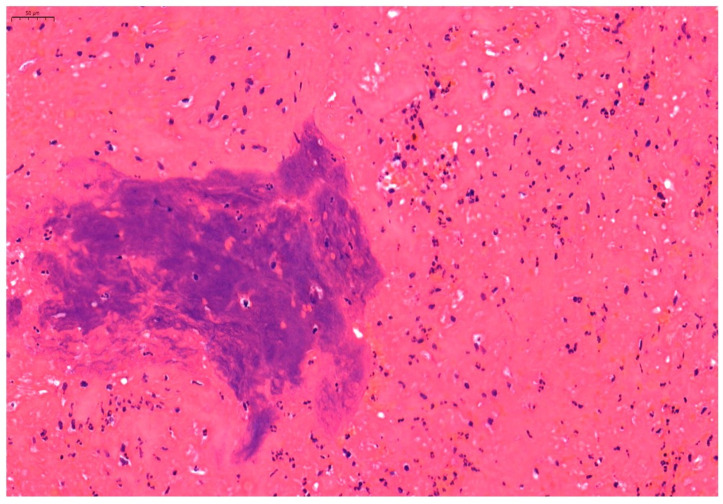

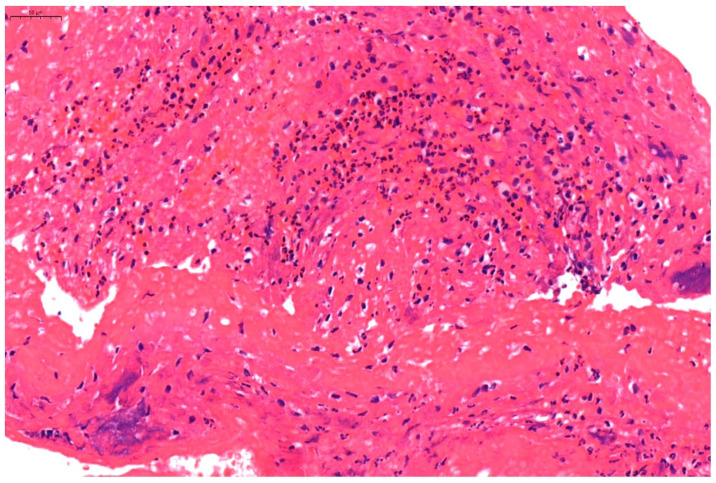
Morphologic analysis of extracted ICD lead vegetation: thrombic masses composed of abundant fibrin fibers, immune cells (predominantly neutrophilic granulocytes), and some bacterial colonies (H + E, light microscopy, 19.3×, 23.1×, and 27.8× magnification, correspondingly).

Considering the elevated risk of embolization and the high Euro SCORE II of 10.9%, the patient was deemed ineligible for surgical intervention. Based on recent data, a minimally invasive percutaneous thrombectomy using the Penumbra aspiration system was preferred for the removal of infectious vegetations. Histological analysis confirmed the presence of necrotic tissue and bacterial colonies (Figure 2).

**Figure 3:**
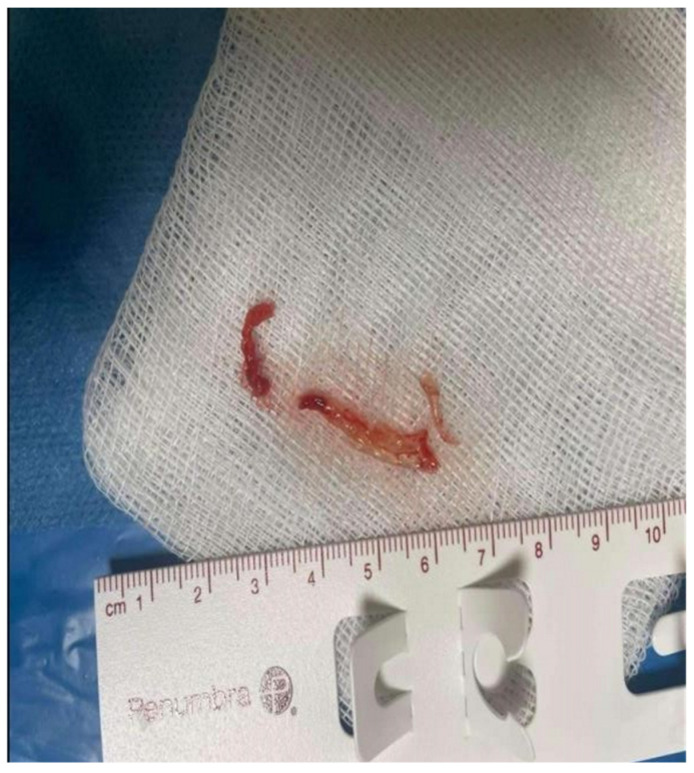
Extracted ICD lead vegetation measuring approximately 3 cm in length.

Following the aspiration of the vegetation, TEE was performed, which revealed small mobile vegetations on the ICD wire (Figure 3).

Following the aspiration of the vegetation, TEE was performed, which revealed small mobile vegetations on the ICD wire (Figure 3). Despite successful partial debulking of the vegetation, the patient’s condition remained complicated, with persistent pain and swelling of the right knee joint, raising concerns regarding the progression of the infection. The patient experienced recurrent fever, and inflammatory markers increased (CRP raised from 5 to 93.2 mg/L). However, blood cultures did not demonstrate bacterial growth. In accordance with the ESC 2023 guidelines on implantable device infections, a decision was made to remove the entire ICD lead. During the surgery, no signs of infection were observed in the surgical site; however, *S. epidermidis* was identified in the samples. Antibiotic therapy with oxacillin was extended for six weeks. After the removal of the ICD lead, the inflammation markers normalized (CRP reduced to 5 mg/L), and the patient was no longer febrile. Infective endocarditis was differentiated from an oncological process. Although PET/CT imaging may provide additional diagnostic value in CIED-related infective endocarditis, particularly in cases with diagnostic uncertainty or suspected septic embolization, it was not performed in the present case. The diagnosis had already been established based on positive blood cultures and echocardiographic findings fulfilling modified Duke criteria. Instead, contrast-enhanced chest CT was performed to exclude malignancy and evaluate possible extracardiac infectious complications. A chest CT scan revealed no free fluid, metastatic masses, or tumors. Additionally, a biopsy of the thyroid gland was conducted, which revealed cystic formation with small calcifications. Recurring infections were differentiated from a urinary tract infection, but urine samples and cultures showed no signs of infection. On 25 April 2024, a new ICD was reimplanted without any complications during or after the procedure. In accordance with ESC guidelines, a prophylactic course of cefazolin was administered, which was subsequently switched to cefuroxime until 3 May 2024. A scheduled ambulatory follow-up on 24 September 2024 revealed optimal parameters of the device, with a battery lifespan exceeding five years and no recorded discharges. In accordance with established recommendations, further monitoring is planned for every one to three years. A chronological summary of the clinical course and management is presented in Table 2.

## 3. Discussion

Infective endocarditis related to cardiac implantable devices is the most severe complication, representing 10% of all infective endocarditis. Removing the entire device is the key to managing these infections. When approaching infected lead removal in cardiac device-related infective endocarditis, a surgical consideration for large (>20 mm) vegetations is recommended. However, a major clinical challenge is that cardiac implantable devices might have been implanted a long time ago in older, comorbid, and fragile patients; thus, combined with a high risk of complications during extraction surgery, cardiac implantable devices can sometimes not be removed. In those cases, patients may require lifelong oral antibiotic suppression treatment, which can decrease their quality of life and increase morbidity and mortality in the short and medium terms. The removal of cardiac implantable electronic devices may also cause septic embolization. Both cardiac implantable device infection and its removal can increase the combined risk of morbidity and mortality and may also present a nidus of reinfection for newly implanted devices [1,17,18].

The annual incidence of cardiac device-related infective endocarditis ranges from 1.5% to 2.5%, generating a significant social and mortality burden. According to the most recent European Heart Rhythm Association guidelines, infected lead extraction is always recommended, and a percutaneous approach is the first choice. Although the rates of successful percutaneous removal are high, in-hospital mortality can reach 2.3%, especially when a systemic infection is suspected. In these settings, as well as in large (>20 mm) vegetations, a vacuum-assisted aspiration of the mass or a conventional surgical approach is suggested. The expansion of percutaneous thrombectomy techniques for the treatment of pulmonary embolism has led to interest in the off-label use of percutaneous mechanical aspiration (PMA) in infective endocarditis that may not be amenable to conventional management strategies. The purpose of PMA is cardiac imaging-guided catheter-based extraction/debulking of vegetations with the goal of enhancing the efficacy of antimicrobial therapy with clearance of bloodstream infections in refractory septicemia, lowering the risk of septic embolization, reducing valve destruction and its hemodynamic consequences, and potentially lessening the hospital length of stay. By delaying or avoiding surgical valve replacement, the risk of prosthesis reinfection may also be decreased. PMA has evolved as an option in the contemporary team-based approach in management of infective endocarditis, and its use in right-sided IE received a class IIb recommendation in the 2023 European Society of Cardiology guidelines for management of infective endocarditis. Furthermore, patients in compounding conditions who are not fit for surgery may benefit from new minimally invasive procedures such as PMA [12,16,19,20,21,22,23,24,25,26].

### 3.1. Treatment Options

Infective endocarditis in clinical practice continues to be a challenging condition. Diagnostic tools and treatment strategies are improving, and the disease is associated with a high risk of death and significant complications. The ESC 2023 guidelines emphasize that the management of IE should be highly personalized and treated from a multidisciplinary perspective [12]. Surgical intervention is needed in about half of IE cases, particularly when complications arise [20]. Severe valve damage leading to heart failure, infections that persist despite proper antibiotics, or recurrent embolic events are common reasons for surgery. Infected prosthetic valves often mandate surgical replacement to prevent further deterioration. Nevertheless, invasive options have a role in selected patients [6]. PMA allows physicians to remove vegetations from heart valves using a catheter-based device, offering an alternative to open-heart surgery. Systems like the Penumbra Indigo have shown encouraging results, especially in patients with large vegetations or implanted cardiac devices [14,25,26,27]. While PMA is not yet standard practice and remains off-label, early clinical experience suggests it may offer a safe and effective alternative when conventional surgery carries prohibitive risk [28,29]. Infective endocarditis treatment options have advantages and disadvantages. Each of the treatments advantages and disadvantages are presented in Table 3.

In our case, empirical antibiotic therapy was initiated and subsequently adjusted to targeted antibiotic therapy according to the antibiotic susceptibility results. According to current guidelines, surgical treatment was recommended; however, considering the patient’s comorbidities, the operative risk was high. Therefore, an off-label innovative treatment approach was chosen, employing the Penumbra aspiration system for the partial debulking of the vegetation. Due to persistent signs of infection and elevated inflammatory markers, removal of the implanted cardiac device leads was undertaken. Following this, the patient’s inflammatory markers normalized, and the treatment was successful. Overall, treating IE demands flexibility and expertise. Antimicrobial treatment remains the first choice, but traditional and innovative surgical options are important, especially in complex cases where traditional surgery is not an option due to excessive risk. With the development of new techniques such as PMA, management options are being extended. Positive effects are already being observed.

### 3.2. Complications

PMA can be associated with vascular injury, blood loss, arrhythmias, and, in rare cases, cardiac perforation. In addition, in cases with no vascular injury, but given the lack of a blood return mechanism with the device used, there can be an average blood loss of 0.5 ± 0.2 L. Blood loss with this device decreases, as the operator experience increases. In addition, there was reported worsening of tricuspid valve (TV) function, increasing regurgitation after the procedure. Some patients underwent TV replacement after clearance of the blood cultures or had subsequent TV surgery. In most cases, the histologic review of retrieved specimens revealed the possibility that the reason for worsening TV regurgitation is a perforation created by the infective endocarditis, as opposed to direct damage related to the aspiration forces. Worsening TR in these interventions is usually tolerated for several weeks and may allow referral to rehabilitation to treat drug addiction before TV surgery [14,30,31].

One of the most serious complications is distal embolization with high mortality. Distal embolization can cause pulmonary embolism, stroke, coronary or peripheral artery embolism. Interventional techniques may be required to retrieve embolized material if clinically indicated. The risk of distal embolization can be drastically decreased with the principles of tailoring device selection with the vegetation characteristics and size, approaching the target while aspiration is on and minimizing contact with the vegetation during large-bore PMA. Moreover, aspiration of a vegetation may lead to transient worsening sepsis, manifested by rigors and fever. In extreme cases, septic shock can occur. Septic shock is an extremely dangerous complication with high mortality rates, and some cases may require extracorporeal membrane oxygenation for shock and respiratory failure. For patients who initially presented with septic shock prior to the procedure, there is an associated increase in mortality. It is also important to avoid upfront PMA prior to the initiation of antimicrobial treatment and to continue this treatment throughout the procedure [14,31,32].

No complications occurred during the described case. Owing to the appropriate choice of treatment strategy, antibiotic therapy, and early intervention, distal embolization was prevented. Significant blood loss during the intervention was also avoided; therefore, blood transfusion was not required. ICD leads were reimplanted after normalization of inflammatory markers and resolution of infection signs, and antibiotic therapy was prophylactically continued; no recurrence of infection was observed. Follow-up echocardiography demonstrated that the TV function remained stable and did not worsen after the interventions. Both prior to infective endocarditis and after completion of its treatment, TV regurgitation remained unchanged at grade I–II.

### 3.3. Case Comparison

The study by Gianni et al. evaluating same-day discharge following uncomplicated transvenous lead extraction (TLE) demonstrated that early discharge may be safe and feasible in carefully selected patients after uncomplicated procedures [33]. However, the study population primarily consisted of clinically stable individuals without major post-procedural concerns, and the findings are not directly generalizable to patients with active systemic infection or large infected vegetations. In contrast, our patient presented with definite cardiac implantable electronic device-related infective endocarditis (CIED-IE), recurrent *Staphylococcus epidermidis* sepsis, a large mobile ICD lead vegetation measuring approximately 3 cm, and multiple comorbidities associated with high operative risk (Euro SCORE II 10.9%). Therefore, unlike uncomplicated TLE cohorts, our case represents a substantially more complex infectious and embolic-risk scenario requiring staged management rather than early discharge. Our case more closely resembles recently published reports describing vacuum-assisted aspiration or percutaneous mechanical aspiration (PMA) for debulking large lead-associated vegetations before definitive extraction. Tarzia et al. described vacuum-assisted removal of large lead vegetations in cardiac device-related infective endocarditis as a less invasive alternative in high-risk patients unsuitable for surgery [17]. Likewise, reports using Penumbra aspiration systems have demonstrated the feasibility of reducing the vegetation burden prior to transvenous lead extraction, particularly in patients with high embolic risk or multiple comorbidities [15,30]. Similar to these reports, a percutaneous Penumbra aspiration strategy was selected in our patient, because conventional surgery was considered prohibitively high risk and the large vegetation size raised significant concerns regarding embolization.

Nevertheless, an important distinction in our case is that aspiration alone did not represent definitive therapy. Despite initial clinical improvement after vegetation debulking, recurrent fever and elevation of inflammatory markers prompted complete ICD lead extraction in accordance with the ESC 2023 recommendations for definite CIED-related infective endocarditis [1]. Following definitive device removal and prolonged targeted antibiotic therapy, the inflammatory markers normalized, no recurrence of infection was observed, and a new ICD was successfully reimplanted. Therefore, our case supports the concept that vacuum-assisted aspiration should be regarded as an adjunctive or bridging strategy rather than a replacement for complete infected device extraction in patients with persistent or recurrent infection. A case comparison summary of infective endocarditis is presented in Table 4.

## 4. Conclusions

Treating device-related endocarditis in elderly patients with multiple comorbidities remains a significant challenge. In selected patients with prohibitive surgical risk, percutaneous vacuum-assisted aspiration may represent a useful adjunctive strategy for reducing the vegetation burden before definitive device extraction. In our case, the Penumbra vacuum system helped control the infection by safely removing large vegetations from the ICD leads. This method was less invasive and allowed us to stabilize the patient before completing the removal of the system. It proved to be a useful bridge between infection control and definitive treatment. Although PMA remains an off-label and non-standardized technique, further clinical experience and prospective studies are needed to better define its role in the management of infective endocarditis, particularly in similar high-risk cases. More clinical experience will help to determine when and how such percutaneous approaches should be used. However, careful patient screening remains paramount.

## Figures and Tables

**Table 1:** Selected laboratory parameters during hospitalization and follow-up.

Parameter	Reference Range	November 2023 (1)	November 2023 (2)	January 2024 (3)	7 February 2024 (4)	8–13 February 2024 (5)	20 February 2024 (8)	23 February 2024 (6)	19 April 2024 (7)	5 February 2025 (9)
**Inflammatory markers**										
CRP (mg/L)	<5	88.1	74.3	79.9	5.0	10.0	93.2	5.0	5.0	5.0
WBC (×10^9^/L)	3.6–10.2	8.8	8.2	11.6	—	6.3	6.1	—	8.9	9.2
Neutrophils, NE (%)	43.5–73.5	66.5	65.2	63.0	—	49.3	63.3	—	69.6	71.7
Procalcitonin (μg/L)	<0.1	—	—	2.79	—	0.60	1.88	—	—	—
**Hematology**										
Hemoglobin (g/L)	135–175	131	137	134	—	129	120	—	158	147
Platelets (×10^9^/L)	152–348	193	227	—	—	—	183	—	221	233
**Renal function**										
Creatinine (μmol/L)	59–104	96	—	155	122	126	127	146	164	123
eGFR (mL/min/1.73 m^2^)	≥60	72.3	—	40.6	54.1	52.1	51.6	43.6	37.9	53.3
**Microbiology**										
Blood culture	Negative	Pos. †	—	—	—	—	—	—	—	—
ICD lead culture	Negative	—	—	—	—	—	—	Pos. ‡	—	—

(1)–(9) Clinical milestones: elbow septic arthritis; oxacillin; sepsis + *S. epidermidis* † + TEE vegetation ~3 cm; Penumbra aspiration; TEE clear; fever relapse; ICD extraction ‡; reimplantation; follow-up. † *S. epidermidis* isolated (oxacillin-sensitive); targeted antibiotic therapy initiated 24 January 2024. ‡ *S. epidermidis* isolated from ICD lead scrapings; oxacillin continued for six weeks post-extraction.

**Table 2:** Chronological timeline of the clinical course and management.

Date	Clinical Course and Management
February 2023	Knee arthroplasty. Right total knee arthroplasty performed for advanced gonarthrotic.
November 2023	Septic arthritis. Hospitalized with left elbow septic arthritis (*S. epidermidis*); treated with oxacillin with temporary improvement.
January 2024	Recurrent infection. Readmission with fever up to 39 °C, elevated CRP (93 mg/L), and leukocytosis. Blood cultures confirmed *S. epidermidis* (oxacillin-sensitive); targeted therapy initiated.
Late January 2024	Echocardiography. TTE identified a large mobile ICD lead vegetation; TEE confirmed ~3 cm vegetation with high embolic risk. Heart Team recommended percutaneous aspiration as bridging strategy.
7 February 2024	Penumbra aspiration. Vacuum-assisted thrombectomy (Penumbra Indigo System, 12F) performed under fluoroscopic guidance; partial vegetation debulking achieved without complications.
8–13 February 2024	Post-aspiration. TEE confirmed no residual vegetation. CRP declined progressively (79 → 5 mg/L); oxacillin continued.
20 February 2024	Fever recurrence. Recurrent fever and CRP rise to 93.2 mg/L: blood cultures negative. Decision made to proceed with complete device extraction per ESC 2023 guidelines.
23 February 2024	ICD lead extraction. Complete ICD system removed under general anesthesia. *S. epidermidis* isolated from lead scrapings; oxacillin continued for six weeks. Wound healed by primary intention.
25 April 2024	ICD reimplantation. After full infection resolution and CRP normalization, new ICD successfully reimplanted without complications.
September 2024–February 2025	Follow-up. Ambulatory reviews confirmed stable device function, normal inflammatory markers, and no infection recurrence.

**Table 3:** Treatment options for infective endocarditis.

Treatment Option	Advantages	Disadvantages/Limitations
Empiric intravenous antibiotic therapy	Immediate treatment; broad pathogen coverage; reduces risk of progression and embolization	Potential overtreatment; nephrotoxicity; may affect microbiological yield
Targeted intravenous antibiotic therapy	Pathogen-specific efficacy; narrower antimicrobial spectrum	Requires prolonged treatment and toxicity monitoring
Vancomycin-based regimens	Effective against MRSA and resistant Gram-positive pathogens	Nephrotoxicity: therapeutic monitoring required
Beta-lactam therapy	Excellent bactericidal activity; preferred for susceptible MSSA	Allergy risk; resistance concerns
Combination antibiotic therapy	Synergistic bactericidal effect in selected cases	Nephrotoxicity and ototoxicity risk
Outpatient parenteral antimicrobial therapy (OPAT)	Reduced hospitalization; improved patient comfort	Catheter-related complications; close follow-up required
Oral step-down antibiotic therapy	Improved convenience; lower healthcare burden	Limited evidence; strict patient selection necessary
Surgical valve repair/replacement	Removes infected tissue; improves outcomes in selected patients	Major operative risk; prosthetic complications
CIED extraction	Essential for eradication of device-related infection	Procedural complications may occur
Supportive therapy	Hemodynamic stabilization and complication management	Does not eradicate infection

**Table 4:** Summary of Case Comparisons.

Characteristic	Our Case	Gianni et al. [33] (Same-Day Discharge After Uncomplicated TLE)	Similar PMA/Penumbra Reports
Clinical setting	Definite CIED-related infective endocarditis	Uncomplicated transvenous lead extraction	High-risk CIED-IE/right-sided endocarditis
Infection status	Active *S. epidermidis* sepsis	Clinically stable; no active systemic infection	Active infection common
Vegetation size	~3 cm mobile ICD lead vegetation	Large vegetations not typical (majority < 10–15 mm)	Usually, >20 mm
Procedural approach	Penumbra aspiration for debulking → staged complete ICD lead extraction	Direct transvenous lead extraction in a single procedure	Aspiration/debulking (PMA/AngioVac) before lead extraction
Indication for aspiration	High embolic risk due to large vegetation; prohibitive surgical risk	Not applicable	Reduce vegetation burden and embolization risk in high-risk patients
Surgical risk profile	High (Euro SCORE II 10.9%)	Selected low/intermediate-risk patients	Frequently high surgical risk
Hospitalization strategy	Prolonged inpatient, staged management	Same-day discharge	Typically inpatient, staged care
Definitive management	Complete device extraction + prolonged targeted antibiotics	Complete extraction during index procedure	Complete extraction after debulking
Guideline alignment	Consistent with ESC 2023 recommendations for definite CIED-IE	Applicable to uncomplicated cases without active infection	Consistent with ESC guidelines as adjunctive/bridge strategy
Outcome	Infection resolved; inflammatory markers normalized; successful ICD reimplantation	Safe same-day discharge; low complication rate	Favorable infection control and clinical improvement reported
Key takeaway	Aspiration useful as a bridge in high-risk CIED-IE but not a substitute for complete extraction	Same-day discharge feasible in carefully selected uncomplicated TLE patients	Aspiration reduces vegetation burden and procedural risk but requires definitive device extraction

## Data Availability

No new data were created or analyzed in this study. Data sharing is not applicable to this article.

## Abbreviations

The following abbreviations are used in this manuscript:

ICD	Implantable Cardioverter-Defibrillator
CIED	Cardiac Implantable Electronic Device
ESC	European Society of Cardiology
CKD	Chronic Kidney Disease
Euro SCORE II	European System for Cardiac Operative Risk Evaluation II
MI	Myocardial Infarction
CABG	Coronary Artery Bypass Grafting
PCI	Percutaneous Coronary Intervention
CRP	C-Reactive Protein
WBC	White Blood Count
MRSA	Methicillin-resistant *Staphylococcus aureus*
eGFR	Estimated Glomerular Filtration Rate
CT	Computed Tomography
TTE	Transthoracic Echocardiography
TEE	Transesophageal Echocardiography
PMA	Percutaneous Mechanical Aspiration
TV	Tricuspid Valve
*S. epidermidis*	*Staphylococcus epidermidis*
